# Long non-coding RNAs in neurodevelopmental disorders

**DOI:** 10.3389/fnmol.2013.00053

**Published:** 2013-12-30

**Authors:** Ilse I. G. M. van de Vondervoort, Peter M. Gordebeke, Nima Khoshab, Paul H. E. Tiesinga, Jan K. Buitelaar, Tamas Kozicz, Armaz Aschrafi, Jeffrey C. Glennon

**Affiliations:** ^1^Department of Cognitive Neuroscience, RadboudUMCNijmegen, Netherlands; ^2^Centre for Neuroscience, Donders Institute for Brain, Cognition, and BehaviorNijmegen, Netherlands; ^3^Department of Neuroinformatics, Radboud UniversityNijmegen, Netherlands; ^4^Department of Anatomy, Radboud UniversityNijmegen, Netherlands

**Keywords:** long non-coding RNA, nervous system development, fragile X syndrome, genomic imprinting, autism spectrum disorders, intellectual disability, schizophrenia

## Abstract

Recent studies have emphasized an important role for long non-coding RNAs (lncRNA) in epigenetic regulation, development, and disease. Despite growing interest in lncRNAs, the mechanisms by which lncRNAs control cellular processes are still elusive. Improved understanding of these mechanisms is critical, because the majority of the mammalian genome is transcribed, in most cases resulting in non-coding RNA products. Recent studies have suggested the involvement of lncRNA in neurobehavioral and neurodevelopmental disorders, highlighting the functional importance of this subclass of brain-enriched RNAs. Impaired expression of lnRNAs has been implicated in several forms of intellectual disability disorders. However, the role of this family of RNAs in cognitive function is largely unknown. Here we provide an overview of recently identified mechanisms of neuronal development involving lncRNAs, and the consequences of lncRNA deregulation for neurodevelopmental disorders.

## INTRODUCTION

Therapeutic strategies for the amelioration of neurobehavioral dysfunction in neurodevelopmental disorders such as intellectual disabilities (ID), or autism spectrum disorders (ASD) are often insufficient for a large patient population. These disorders have complex behavioral and cognitive phenotypes that are thought to develop through disturbances in neural circuitry and synaptic function. Moreover, genetic epidemiology and population genetic studies suggested that a spectrum of allelic risk underlies complex traits like ID (Geschwind, 2008). However, the existence of risk alleles rarely confers diagnostic specificity (Hitzemann et al., 2013). One possible explanation for this may involve dysregulation of the rate of gene transcription/translation by small or long non-coding (nc)RNAs, leading to abnormal expression of ID-risk genes of phenotypic relevance (Olde Loohuis et al., 2012). Several studies have now indicated altered levels of brain-specific small and long ncRNA in ID and other neurodevelopmental disorders (Willemsen et al., 2011). lncRNAs constitute a large fraction of the total ncRNA pool, each exceeding 200 nucleotides in length. It was initially assumed that lncRNAs merely act as primary or precursor transcripts for the production of short ncRNAs (sncRNAs) such as microRNAs (miRNAs) or small nucleolar RNAs (snoRNAs; Aschrafi et al., 2008). Conversely to snoRNAs genes, however, the evolutionary conservation of lncRNAs often extends beyond the overlapping sncRNA segments (Wang et al., 2004). They have been shown to either act solely, or together with proteins, exerting a wide range of cellular roles, e.g., their regulation of transcription and RNA processing (Wang et al., 2008). The purpose of this review is to emphasize the role of lncRNAs in regulating neuronal molecular pathways, and to highlight their putative role in dysregulation of these mechanisms in neurodevelopmental disorders.

## MECHANISMS OF ACTION OF lncRNAs

### LncRNA TRANSCRIPTION MODULATES THE EXPRESSION OF OTHER GENES

**FIGURE 1:**
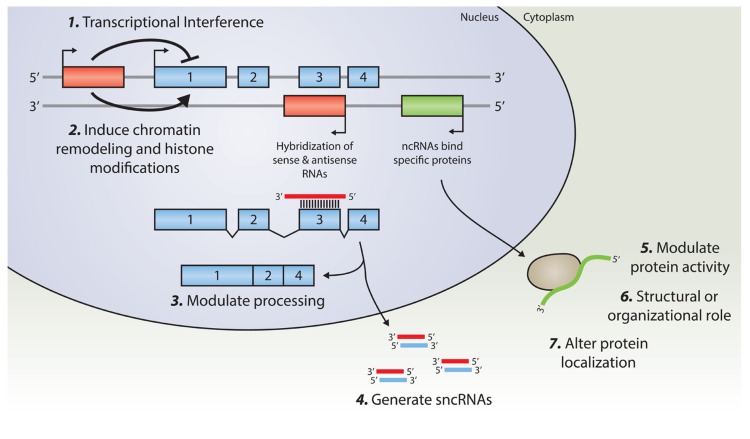
**An overview of known regulatory mechanisms for lncRNA.** Transcription from an upstream promoter can negatively or positively affect the expression of a downstream gene via (1) transcriptional interference mediated by inhibition of RNA Polymerase 2 recruitment, or by (2) inducing chromatin remodeling and histone modification. Alternatively, an antisense transcript is able to hybridize to the overlapping sense transcript and modulate further processing (3), or provide a substrate for Dicer, or other nucleases, in order to generate various small non-coding RNAs (4). By binding to specific protein partners, a long noncoding transcript may modulate the activity of that particular protein (5), serve as a structural component that allows the formation of a larger RNA–protein complex (6), or alter the cellular localization of the protein (7).

Transcription of lncRNAs from alternative transcription start sites in the vicinity of other genes may interfere with the transcription efficiency of that gene (Martens et al., 2005; Martianov et al., 2007). These transcriptional interference mechanisms have been shown to regulate key developmental pathways, such as those involving *Hox*-genes expression (Wang et al., 2011). A complete overview of potential regulatory mechanisms of lncRNAs is provided in (Guttman and Rinn, 2012) or (Ponting et al., 2009). A schematic overview of lncRNAs cellular function is depicted in **Figure 1**.

### LncRNAs MAY REGULATE RNA-PROCESSING AND PROTEIN ACTIVITY

Initial research suggested that the functions of lncRNAs relate to their interactions with the RNA-binding proteins (RBPs), a protein family highly abundant in the brain (Smart et al., 2007). Due to the long sequence and structural characteristics of lncRNAs, along with various RBPs and RNA-binding domains, numerous combinations of lncRNA/RNA-binding proteins can be formed. This allows the recruitment of various protein-complexes and a multitude of “downstream” functions. Previous studies suggested that lncRNAs, in concert with RBPs and different protein-complexes, have the capacity to induce chromatin remodeling and histone modification, as well as modulating alternative splicing and protein-activity. For example, *Alu RNA* and *B2 RNA* may directly affect RNA polymerase II activity. Both are transcribed from Short Interspersed Nuclear Elements (SINEs; Yakovchuk et al., 2009). *Alu RNA* and *B2 RNA* block binding of RNA polymerase II to the promoter and change the conformation of the transcription initiation complex significantly.

### CHROMATIN REMODELING AND HISTONE MODIFICATION CAN BE INDUCED BY lncRNAs

LncRNAs are capable of mediating the activity of proteins involved in chromatin remodeling and histone modification, including those at the Polycomb Repressive Complex 2 (PRC2) complex (Khalil et al., 2009; Tsai et al., 2010) and the CBP/p300 complex (Wang et al., 2008). A genome-wide study revealed that approximately one third of conserved intergenic lncRNAs associates with either the PRC2 complex or the CoREST/REST or SCMX proteins, all known chromatin-modifying proteins (Khalil et al., 2009). A prominent epigenetic mechanism exerted by lncRNAs is the X-chromosome inactivation. The extent of this control is unique among the chromosomes and is disrupted in X-linked IDs. X-chromosome inactivation is mediated via the lncRNA *Xist* that binds to one of the X-chromosomes (Zhao et al., 2008). *RepA* was found to be both part of the *Xist* lncRNA, as well as expressed by itself (Zhao et al., 2008). The *RepA* lncRNA is able to bind the histone methyltransferase Enhancer of Zester Homolog 2 (Ezh2), which is a subunit of the PRC2. The recruitment of the PRC2 complex by *Xist*, via the *RepA* sequence, allows trimethylation on lysine-27 of H3 histones (H3K27), effectively repressing gene expression, and inactivating the X-chromosome (Zhao et al., 2008). Very Recently, *Xist* was found to function in a two-step mechanism, though targeting of gene-rich islands before gene-poor domains (Simon et al., 2013).

## FUNCTIONAL ROLES OF LncRNAs IN NERVOUS SYSTEM DEVELOPMENT AND FUNCTION

Multiple lines of evidence suggest that dysregulated processes as seen in neurodevelopmental disorders are based on mechanisms that are under tight regulation by lncRNAs (see below). A number of ncRNAs were found to be specifically expressed within the hippocampus (Mercer et al., 2008b), a region involved in processing and consolidation of memories. Several lncRNAs originate from genomic regions associated with protein-coding genes involved in memory formation and maintenance, such as an lncRNA transcribed antisense to *Camkk1*, which is involved in male-specific memory formation (Mercer et al., 2008a).

During brain development, differentiation of neural stem cells and progenitors is crucial. Recently, various lncRNAs have been linked to these events, implying a key role for lncRNAs not only during development, but also in several neuropathologies (reviewed by e.g., Qureshi et al., 2010). For example, a subset of lncRNAs are specifically associated with genes from the *Dlx*-family, known to be involved in brain development in mammals and Drosophila. Two of the differentially expressed lncRNAs, *Evx1as* and *Hox5b/6as* were shown to be associated with trimethylated H3K4 histones and histone methyltransferases (Dinger et al., 2008). In addition, embryonic ventral forebrain-2 (*Evf2*) is transcribed from the *Dlx5/Dlx6* locus, antisense to the *Dlx6* gene (Feng et al., 2006). *Dlx6* is a homeobox-containing transcription factor important in forebrain neurogenesis (Stenman et al., 2003). Furthermore, 659 evolutionary conserved murine lncRNAs have been identified of which the brain-specific lncRNAs are preferentially (2 to 3-fold increase) located adjacent to brain-expressed protein-coding genes, involved in transcriptional regulation, or in nervous system development (Ponjavic et al., 2009).

Recent studies identified 945 lncRNAs, of which 174 were differentially expressed in the mouse embryoid bodies; and that are annotated to developmentally important events relating to stem cell pluripotency (Dinger et al., 2008). One of these RNAs, *Sox2OT* (*Sox2* Overlapping Transcript) is a highly conserved lncRNA that overlaps the *Sox2* gene (Fantes et al., 2003). *Sox2* is a transcription-factor critical in maintaining self-renewal properties of neural stem cells (Mizuseki et al., 1998). Similar to *Sox2, Sox2OT* is present in neural stem cells and is downregulated during differentiation (Amaral et al., 2009).

During fate-specification from neuronal oligodendrocyte bipotent progenitors into GABAergic interneurons, 56 lncRNAs were found to be upregulated, including *Gtl2, Rian, Evf2* and *Copg2a*s, but also the novel *AK044422* (Mercer et al., 2010). Interestingly, *AK044422* overlaps with miR-124a, a highly conserved and highly expressed brain-specific miRNA previously implicated in regulating neuronal specification and differentiation (Makeyev et al., 2007; Visvanathan et al., 2007). Synaptogenesis is a pivotal process during neuronal development, which is altered in various neurodevelopmental disorders (reviewed by e.g., (Zoghbi, 2003; Ecker et al., 2013)). Metastasis-associated lung adenocarcinoma transcript 1 (*Malat1*) is an lncRNA that is enriched in nuclear speckles (Hutchinson et al., 2007; Clemson et al., 2009). There, it co-localizes with splicing factors to controls the expression of genes involved in synapse function and synaptogenesis (Bernard et al., 2010).

## LncRNAs ARE INVOLVED IN NEURODEVELOPMENTAL DISORDERS

Several lncRNAs are either differentially expressed in or associated with neurodevelopmental disorders, such as Prader–Willi syndrome (PWS), Angelman syndrome (AS), ID, and ASD (**Table 1**). The role of lncRNAs is possibly best understood in genomic imprinting disorders such as PWS (Wevrick and Francke, 1997; Jong et al., 1999) and AS (Runte et al., 2004), both of which feature learning difficulties but otherwise have different symptoms (further discussed below).

**Table 1:** An overview of the lncRNAs identified in neurodevelopmental disorders.

Disorder	LncRNA	Significance	Reference
PWS	*SNORD116 (HBII-85)* C/D box cluster	Microdeletions including this cluster cause PWS (phenotype)	Duker et al. (2010), Sahoo et al. (2008), de Smith et al. (2009)
	*IPW*	Not expressed in PWS	Wevrick et al. (1994)
	*ZNF127AS*	Disrupted expression in PWS	Jong et al. (1999)
AS	*UBE3A-AS*	Increased or decreased expression in AS	Runte et al. (2004)
FXS	*FRM4 (FMR1-AS1)*	Silenced in FXS patients; knockdown results in alterations in cell cycle regulation and increased apoptotic cell death	Ladd et al. (2007), Khalil et al. (2008)
	*BC1*	Associated with fragile X syndrome	Zalfa et al. (2003, 2005)
Rett syndrome	AK087060 AK081227	Upregulated in MECP2 KO mice; AK087060 associated with the downregulation of its host gene, GABA receptor subunit Rho 2 (Gabbr2)	Petazzi et al. (2013)
DS	*NRON*	Regulates nuclear shutting of NFAT, whose reduced activity leads to DS features	Willingham et al. (2005), Arron et al. (2006)
2p15-p16.1 microdeletion syndrome	*FLJ16341*	In critical region with three protein-coding genes: BCL11A, PAPOLG, and REL	Hancarova et al. (2013)
MCOPS3	*SOX2OT*	Modulates expression of SOX2, in which genetic defects cause micropthalmia syndrome 3.	Fantes et al. (2003), Amaral et al. (2009)
ASD	*ST7OT1*	Associated with autism in one patient	Vincent et al. (2002)
	*ST7OT2*		
	*ST7OT3*		
	*PTCHD1AS1; PTCHD1AS2; PTCHD1AS3*	Deletions are only found in males with ASD and not in male control individuals.	Noor et al. (2010)

*The disorders are listed in the first column (PWS, Prader–Willi syndrome; AS, Angelmann syndrome; FXS, fragile X syndrome; DS, down syndrome; MCOPS3, micropthalmia syndrome 3; ASD, autism spectrum disorder).*

### IMPRINTING DISORDERS

Genomic imprinting is mediated by various processes such as DNA methylation and histone modification, but also by ncRNAs (Bartolomei, 2009). PWS (MIM 176270) is characterized by infantile hypotonia, early childhood obesity, short stature, hypogenitalism/hypogonadism, ID, and other behavioral problems including temper tantrums. The genetic cause of the disorder lies in a disruption of the paternal chromosome 15q11.2q13, since the maternal chromosome is inactive through imprinting (Horsthemke and Wagstaff, 2008). To date, two genes have functionally been associated with the pathology of the disorder: *NECDIN* and small nuclear ribonucleoprotein polypeptide N *(SNRPN)*. *Necdin* deficient mice show a subset of the multiple clinical manifestations of PWS (Muscatelli et al., 2000). *SNRPN* encodes the SmN splicing factor, the SNRPN upstream reading frame (*SNURF*) and partially overlaps the *UBE3A* gene. Importantly, the downstream introns of *SNRPN* contain C/D box-containing *SNORD116* (*HBII-85*) snoRNA clusters whose expression is under control of the *SNRPN* promoter (Runte et al., 2001). Several case reports indicated that paternally inherited microdeletions of this cluster cause PWS (Sahoo et al., 2008; de Smith et al., 2009; Duker et al., 2010). Moreover, two mouse models with targeted deletions in the *MBII-85* snoRNA cluster exhibited a similar phenotype as other PWS models, which included decreased activity, hypotonia at birth, and postnatal growth retardation (Skryabin et al., 2007; Ding et al., 2008).

*IPW* (Imprinted gene in the PWS region) is located in the proximal chromosome 15q, merely 150 kb distal to *SNRPN* and is not expressed in patients with 15q11-q13 deletions (Wevrick et al., 1994). Additionally, *ZNF127* is located in the same region and has been reported to have a disrupted expression in PWS. This gene has a potentially non-coding antisense gene, *ZNF127AS*, which might be regulating the imprinting of *ZNF127* gene (Jong et al., 1999).

Angelman syndrome (MIM 105830) is caused by a disruption of the maternal allele of chromosome 15q11-q13, covering the same genomic location as PWS. However, the symptoms are different and include intellectual disability, movement or balance disorder, typical abnormal behaviors, and severe limitations in speech and language. The genetic underpinning of the disorder is thought to be a disruption in the *UBE3A* gene (Matsuura et al., 1997). The *UBE3A-AS* gene is transcribed antisense to the *UBE3A* gene and repression of *UBE3A* is dependent on *UBE3A-AS* (Chamberlain and Brannan, 2001; Johnstone et al., 2006). However, another study suggests that silencing of the paternal *UBE3A* can also occur when *UBE3A-AS* is not present, indicating that the regulation is more complex (Le Meur et al., 2005).

### INTELLECTUAL DISABILITY

Despite the highly variable genetic etiology in ID, only a limited number of molecular and cellular pathways appear to be affected by the magnitude of different gene mutations. ID genes have been shown to cluster in pathways underlying neurogenesis, neural migration, neuronal outgrowth, and synaptic function (van Bokhoven, 2011). Numerous studies have suggested that synaptogenesis and normal synaptic function is dependent on the activity of a large number of proteins, and that disturbance of individual components within the network, or alterations of their activities causes synaptic dysfunction, phenotypically culminating in ID (Aschrafi et al., 2005). Regulation of gene transcripts by small and large ncRNAs may underlie epigenetic control of synaptic activity in ID and other neurodevelopmental disorders. Previous studies have indicated that disruption of lncRNA expression and signaling impairs synaptic plasticity, and results in severe cognitive impairment in mice, and human, which are detailed below.

#### Fragile X Syndrome

Fragile X syndrome (FXS, MIM 300624) is inherited via an X-linked dominant pattern and characterized by moderate to severe mental retardation, macro-orchidism, and distinct facial features. The disorder is caused by an unstable expansion of a CGG repeat in the *FMR1* gene leading to silencing of the gene by methylation of repeat and promoter (Sutcliffe et al., 1992), resulting in decreased FMRP protein levels in the brain (Devys et al., 1993). Accumulating evidence suggests that the etiology of the disorder is influenced by lncRNAs. The promoter of *FMR1* is bidirectional and can also give rise to the lncRNA *FMR4* or *FMR1-AS1*, a gene transcribed in the antisense orientation and overlaps the CGG repeat region. *FMR4* is similar to *FRM1* in being silenced in FXS patients and upregulated in permutation carriers (Ladd et al., 2007; Khalil et al., 2008). Following siRNA knockdown of *FMR4*, alterations in cell cycle and apoptosis were reported. Conversely, overexpression of *FMR4* resulted in increased cell proliferation. Additionally, knockdown of *FMR4* did not influence *FMR1* expression and vice versa, suggesting an independent mechanism from *FMR1* (Khalil et al., 2008). Together, these findings points toward a contribution of *FMR4* in the pathology of FXS.

Recently, Pastori et al. (2013)** discovered two new transcripts in the *FMR1* gene locus: *FMR5* and *FMR6. FMR5* was similarly expressed in brain regions from unaffected and permutation individuals and full mutation patients, whereas *FMR6* was silenced in full mutation and permutation carriers. According to the authors, this might suggest an abnormal transcription or chromatin remodeling prior to transition to the full mutation. In addition to the finding that both *FMR5* and *FMR6* are expressed in blood leukocytes, these lncRNAs are potentially useful as biomarkers in FXS.

FMRP, the protein that is encoded by *FMR1*, acts as a translational repressor of specific mRNAs at the synapse and associates with the dendritic RNA *BC1* (Zalfa et al., 2003). *BC1* enables the interaction of FMRP with the target mRNAs; and FMRP can directly bind to *BC1* and its human analog *BC200* via its N-terminus. Of note, the 5′ stem loop of *BC1* is involved in FMRP recognition and this region is complementary to FMRP target mRNAs (Zalfa et al., 2005). Taken together, the studies suggested that *BC1* is a ncRNA that is essential for the repression of mRNAs via FMRP and loss of this repression in FXS patients could result in synaptic dysfunction. It should be noted that, In Iacoangeli et al. (2008), five independent groups reported that results published by Zalfa et al. (2003) are not reproducible. Thus, there is no confirmation, independent of the Bagni group, of a specific physical link between FMRP and BC1 RNA.

#### Rett syndrome

Rett syndrome (MIM 312750) is characterized by arrested development between 6 and 18 months of age in females, regression of acquired skills, loss of speech, stereotypical movements, seizures, and ID. Mutations in the *MECP2*, which binds methylated CpGs and can both activate and repress transcription, were first described to be the cause of the disorder (Amir et al., 1999). While assessing the transcriptome of male *Mecp2* hemizygous knockout mouse brains (Petazzi et al., 2013), it was revealed that the lncRNAs *AK081227* and *AK087060* were both significantly upregulated as compared to wild-type littermates. Importantly, overexpression of *AK08127* was associated with the downregulation of its host coding protein gene, the gamma-aminobutyric acid receptor subunit Rho 2. This suggest that transcriptional dysregulation of lncRNAs may have the capacity to contribute to the etiology of Rett syndrome.

#### Down syndrome

**FIGURE 2:**
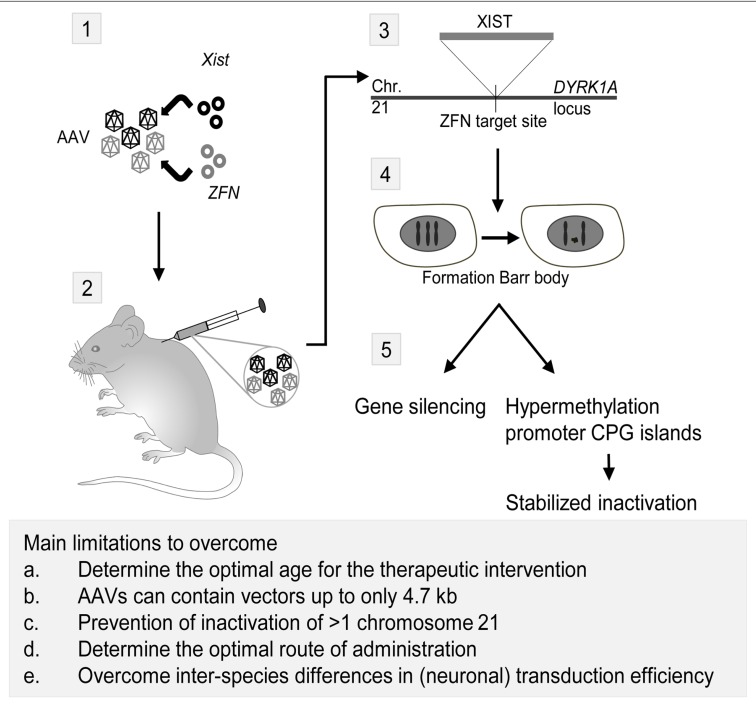
**Proposed strategy for a therapeutic application of *Xist* and zinc finger nucleases (ZFN) to treat trisomy 21.** Adeno-associated viruses (AAVs) are currently the most promising CNS gene delivery vector (for review, see Gray, 2013). As shown in this scheme, the first step in the approach would be incorporation of plasmids containing *Xist* and ZFN targeted to the *DYRK1A* locus on chromosome 21 in AAVs (1). Next, injection of the viruses in rodents can be performed intracranial, intravenous or in the cerebrospinal fluid (2). Intracranial injections have been successfully performed in mammals as large as cats, but an estimated number of 20–30 required injections per hemisphere in human infants rendered this technique unfavorable over alternatives (Vite et al., 2005). AAV9 vectors have the capacity to cross the blood-brain barrier and transduce neurons and astrocytes (Foust et al., 2009), making intravascular injection of viral vectors for CNS targeted gene therapy a possibility. The third possible route of administration is injecting the viral vectors in the cerebrospinal fluid (CSF), thus transducing the central nervous system effectively even in non-human primates (Samaranch et al., 2012). After injection of the AAVs and transduction of the viruses in the cells, *Xist* will be incorporated on the ZFN target site in the *DYRK1A* locus on chromosome 21 (3). Here, it will induce the formation of a chromosome 21 Barr body (4), which will lead to gene silencing and hypermethylation of promoter CPG islands, effectively stabilizing the inactivation of the chromosome (5; Jiang et al., 2013). Eventually, this approach may lead to a CNS-wide inactivation of the third chromosome 21, thereby reducing the symptoms of the trisomy 21 disorder. However, several major limitations have to be overcome in order to translate this proposed approach to humans. First, the optimal age for the therapeutic intervention should be established. The majority of the *in vivo* gene therapy studies have been performed in juvenile or adult animals, but starting the treatment at an earlier age should be considered in order to achieve the best therapeutic effect. Moreover, the optimal route of delivery for CNS gene therapy is currently not established yet, with possibilities being intravascular injection, injection in the CSF, and to a lesser extend, intracranial injections. Third, a practical issue of using AAVs in the therapeutic approach is the limitation of AAVs to contain vectors up to only 4.7 kb in length. This is insufficient for the *Xist* containing vector used in the proof-of-principle study by Jiang et al. (2013). Last, comparing intravascular injection of vectors with a CNS target revealed that both neuronal and overall transduction efficiency in primates is considerably lower than in rodents, the latter most likely due to circulating pre-existing neutralizing AAV antibodies (Gray et al., 2011).

Down syndrome (DS) or Trisomy 21 (MIM 190685) is characterized by ID, distinct facial characteristics and congenital heart defects. The lncRNA *NRON* may be involved in DS, since *NRON* modulates cytoplasmic-to-nuclear transport of NFAT (Willingham et al., 2005). Decreased nuclear NFAT activity leads to DS-like characteristics in animal models, suggesting a possible role for *NRON* in DS (Arron et al., 2006). Recently, an inducible *XIST* was introduced on chromosome 21 using genome editing (Jiang et al., 2013). This approach created a model to investigate genomic expression changes and cellular pathologies of trisomy 21. Notably, deficits in proliferation and neural rosette formation are rapidly reversed upon silencing one chromosome 21, representing a major step toward potential development of “chromosome therapy” (see **Figure 2** for a proposed approach).

#### Other syndromic neurodevelopmental disorders

In the last decade, several new rare microdeletion syndromes were identified. One of these is the 2p15-p16.1 microdeletion syndrome (Rajcan-Separovic et al., 2007), characterized by ID, autistic features, microcephaly, short stature, and various dysmorphic facial features. The genomic cause of this disorder remains to be elucidated, but the susceptibility candidate genes include *BCL11A*, *PAPOLG* and *REL* and one lncRNA gene *FLJ16341*, although the function of this lncRNA is still elusive.

### AUTISM SPECTRUM DISORDER

Autism spectrum disorders is an umbrella term for various developmental disorders, including autism, pervasive developmental disorder not otherwise specified (PDD-NOS) and the Asperger syndrome. Common symptoms of the various ASD disorders include problems of reciprocal social interactions, verbal and non-verbal communication, and rigid and stereotyped behaviors. ASD is a clinically and etiologically heterogeneous disorder with a complex genetic architecture. Not only multiple common genetic variants appear to be involved, each with small effect size, but also rare variants with strong effect size (Devlin and Scherer, 2012). The latter are mostly *de novo* mutations, as evidenced by whole-exome and genome sequencing studies in ASD patients (Talkowski et al., 2012; Vulto-van Silfhout et al., 2013), or copy number variations (CNVs; Poelmans et al., 2013). Microarray analysis shows that 5–10% of subjects with ASD have an identifiable genetic etiology in recurrent or *de novo* chromosomal rearrangements (Marshall et al., 2008). In the last decade, several studies reported aberrant expression of lncRNAs, suggesting that these might be important in the etiology of the disorder. Recently, Ziats and Rennert (2013) showed that over 200 lncRNAs were differentially expressed in a microarray of postmortem prefrontal cortex and cerebellum tissue of ASD patients. A decade earlier, Vincent et al. (2002) identified a novel autism locus, which includes the gene *RAY1/ST7*. This locus contains at least four non-coding genes (*ST7OT1-4*), both on the sense and antisense strands that potentially regulate *RAY1/ST7*. Several rare variants were detected in autism patients on either the *RAY1/ST7* or the *ST7OT1-3* genes that were not observed in a control population.

Mutations in the X-chromosome *PTCHD1* gene have been reported to involve X-linked ID and ASD (Noor et al., 2010; Filges et al., 2011). Although the exact function of the gene is still unknown, several lines of evidence suggest that it might have a causative role in a subset of ID and/or ASD patients (Filges et al., 2011). On the antisense strand of the *PTCHD1* gene, several overlapping lncRNAs (*PTCHD1AS1, PTCHD1AS2* and *PTCHD1AS3*) were detected, which may serve as regulators for *PTCHD1*, since the 5′ exons are adjacent on opposite strands.

## CONCLUSION

Regulation of epigenetics processes during brain development and in activity-dependent brain functions are key to the symptomology underlying many neurodevelopmental disorders. In recent time, a wide range of cutting-edge “omics” and bioinformatics based technologies vastly accelerated our understanding of the key molecular players and mechanisms involved in regulating these epigenetic processes. In contrast to the earlier held view that lncRNAs were merely transcriptional noise, it is now apparent that lncRNAs exert important regulatory functions in the brain, both during adult and developmental stages and represent a key epigenetic mediator of these processes. The interplay between lncRNAs and chromatin remodeling factors may be key to understanding the role of epigenetics in neurodevelopmental disorders (Kramer and van Bokhoven, 2009). LncRNAs are now believed to modulate molecular events during neurogenesis, cell-fate decisions, differentiation and maturation, but are also involved in higher brain functions such as memory formation. The large number of brain-expressed lncRNAs suggests that many more such higher-order functions might also be modulated by lncRNA-mediated mechanisms, which remain to be more fully illustrated in future research efforts. Animal models of lncRNA function, e.g. knockout mice for *Malat1* (Zhang et al., 2012) and *Neat1* (Nakagawa et al., 2011), have been developed recently and might provide a better insight in lncRNA-mediated mechanisms. However, already at this stage it is clear that lncRNAs may offer a unique approach to modulate pathogenetic events in the causation of neurodevelopmental disorders.

## Conflict of Interest Statement

The authors declare that the research was conducted in the absence of any commercial or financial relationships that could be construed as a potential conflict of interest.

## ACKNOWLEDGMENTS

The research of the authors is supported by fundings from the European Community’s Seventh Framework Programme (FP7/2007-2013) under grant agreement no. 278948, and the Marie Curie International Reintegration Grant.
